# Supplementary material for: Y chromosome sequence and epigenomic reconstruction across human populations
Source: Commun Biol. 2023 Jun 9;6:623. doi: 10.1038/s42003-023-05004-9 (PMC10256797; doi:10.1038/s42003-023-05004-9)
Supplement: Supplementary file 3 — Description of Additional Supplementary Files [file 42003_2023_5004_MOESM3_ESM.pdf]

Description of Additional Supplementary Files

File name: Supplementary Data 1
Description: Sequencing data metrics.

File name: Supplementary Data 2
Description: Chromosome enrichment and coverage calculations per haplogroup.

File name: Supplementary Data 3
Description: Assembly metrics overview.

File name: Supplementary Data 4
Description: Percentage of sequence aligning between T2T Y chr assembly, GRCh38 Y chr and Y chr of the present study. Repetitive content of each assembly.

File name: Supplementary Data 5
Description: *Sniffles v2* structural variants detected using ONT data for each Y chromosome haplogroup data mapped to the GRCh38 reference chromosome Y assembly.

File name: Supplementary Data 6
Description: Table with amount of indels harboring repetitive elements (REs) and proportion of indels with high content of REs. The table also reports how many variants contain different types of REs.

File name: Supplementary Data 7
Description: *Assemblytics* results looking for structural variants using assemblies of each Y chromosome cell line.

File name: Supplementary Data 8
Description: The source data behind the graphs in the paper.
