# Supplementary material for: Y chromosome sequence and epigenomic reconstruction across human populations
Source: Commun Biol. 2023 Jun 9;6:623. doi: 10.1038/s42003-023-05004-9 (PMC10256797; doi:10.1038/s42003-023-05004-9)
Supplement: Supplementary file 5 — Reporting Summary [file 42003_2023_5004_MOESM5_ESM.pdf]

nature portfolio

Corresponding author(s): Paula Esteller-Cucala
Tomas Marques-Bonet

Last updated by author(s): May 29, 2023

Reporting Summary

Nature Portfolio wishes to improve the reproducibility of the work that we publish. This form provides structure for consistency and transparency in reporting. For further information on Nature Portfolio policies, see our Editorial Policies and the Editorial Policy Checklist.

Statistics

For all statistical analyses, confirm that the following items are present in the figure legend, table legend, main text, or Methods section.

n/a	Confirmed	
☐	☒	The exact sample size (*n*) for each experimental group/condition, given as a discrete number and unit of measurement
☐	☒	A statement on whether measurements were taken from distinct samples or whether the same sample was measured repeatedly
☐	☒	The statistical test(s) used AND whether they are one- or two-sided<br>*Only common tests should be described solely by name; describe more complex techniques in the Methods section.*
☐	☒	A description of all covariates tested
☐	☒	A description of any assumptions or corrections, such as tests of normality and adjustment for multiple comparisons
☐	☒	A full description of the statistical parameters including central tendency (e.g. means) or other basic estimates (e.g. regression coefficient) AND variation (e.g. standard deviation) or associated estimates of uncertainty (e.g. confidence intervals)
☐	☒	For null hypothesis testing, the test statistic (e.g. *F*, *t*, *r*) with confidence intervals, effect sizes, degrees of freedom and *P* value noted<br>*Give P values as exact values whenever suitable.*
☒	☐	For Bayesian analysis, information on the choice of priors and Markov chain Monte Carlo settings
☐	☒	For hierarchical and complex designs, identification of the appropriate level for tests and full reporting of outcomes
☐	☒	Estimates of effect sizes (e.g. Cohen's *d*, Pearson's *r*), indicating how they were calculated

Our web collection on statistics for biologists contains articles on many of the points above.

Software and code

Policy information about availability of computer code

Data collection	A description of all software used is provided in the manuscript.<br>Software used for data collection:<br>MinKNOW v21.02-beta4~xenial.
Data analysis	A description of all software used is provided in the manuscript.<br>Software used for data analysis<br>minimap2 v2.17-r941<br>SAMTOOLS v1.12<br>Flye v2.9<br>Guppy v5<br>Racon v1.3.1<br>minimap2 v2.9-r720<br>medaka v1.4.1<br>HyPo v1.0.3<br>purge_dups v1.2.5<br>minimap2 v2.14-r883<br>Filtlong v0.2.0<br>MuMmer v3.23<br>Sniffles v2.0.2

SAMTOOLS v1.9
SURVIVOR v1.0.7
Assemblytics v1.2.1
RepeatMasker v4.1.2-p1
graphtyper v2.7.5
nanopolish v0.13.2
R v3.6.0
preprocessCore v1.56.0
annotatr v1.24.0

For manuscripts utilizing custom algorithms or software that are central to the research but not yet described in published literature, software must be made available to editors and reviewers. We strongly encourage code deposition in a community repository (e.g. GitHub). See the Nature Portfolio guidelines for submitting code & software for further information.

Data

Policy information about availability of data

All manuscripts must include a data availability statement. This statement should provide the following information, where applicable:

- Accession codes, unique identifiers, or web links for publicly available datasets
- A description of any restrictions on data availability
- For clinical datasets or third party data, please ensure that the statement adheres to our policy

All sequencing data generated for this study has been deposited at the European Nucleotide Archive (ENA) under the study accession PRJEB58141. Assemblies are deposited at the ENA under the study accession PRJEB59245. Raw sequencing data for the A0 haplogroup (cell line HG02982) were retrieved from ENA study accession PRJEB28143 and its assembly from the accession ULGL01000000. The source data underlying Figs. 1, 2 and 3 are provided in Supplementary Data 8. All other relevant data are available upon request.

Human research participants

Policy information about studies involving human research participants and Sex and Gender in Research.

Reporting on sex and gender	For the study of Y chromosome, only male samples were used.
Population characteristics	Population group and haplotype belonging to each sample is stated along the text (particularly Figure 1A and Supplementary Table 1).
Recruitment	Samples were recruited accounting for population representation.
Ethics oversight	Not applicable.

Note that full information on the approval of the study protocol must also be provided in the manuscript.

Field-specific reporting

Please select the one below that is the best fit for your research. If you are not sure, read the appropriate sections before making your selection.

☒ Life sciences ☐ Behavioural & social sciences ☐ Ecological, evolutionary & environmental sciences

For a reference copy of the document with all sections, see nature.com/documents/nr-reporting-summary-flat.pdf

Life sciences study design

All studies must disclose on these points even when the disclosure is negative.

Sample size	We used seven samples for our study: one publicly available and six newly generated in this study.
Data exclusions	None reported.
Replication	Replication was not required in most cases. For adaptive sampling data generation, two runs of the same cell line were generated.
Randomization	Randomization was not required.
Blinding	No blinding was required for this study.

Reporting for specific materials, systems and methods

We require information from authors about some types of materials, experimental systems and methods used in many studies. Here, indicate whether each material, system or method listed is relevant to your study. If you are not sure if a list item applies to your research, read the appropriate section before selecting a response.

Materials & experimental systems

n/a	Involved in the study
☒	☐ Antibodies
☐	☒ Eukaryotic cell lines
☒	☐ Palaeontology and archaeology
☒	☐ Animals and other organisms
☒	☐ Clinical data
☒	☐ Dual use research of concern

Methods

n/a	Involved in the study
☒	☐ ChIP-seq
☒	☐ Flow cytometry
☒	☐ MRI-based neuroimaging

Eukaryotic cell lines

Policy information about cell lines and Sex and Gender in Research

Cell line source(s)	All cell lines were lymphoblastoid cell lines purchased from the Coriell Institute.
Authentication	The cell lines are authenticated at the source (Coriell), see https://www.coriell.org/0/pdf/CC_Process_Flow.pdf . The cell lines were sequenced.
Mycoplasma contamination	Commercial cell lines were not tested for mycoplasma contamination after being purchased, but contamination would be detectable though genome sequence analysis.
Commonly misidentified lines (See ICLAC register)	No commonly misidentified cell lines were used in the study.
